# Retraction Note to: The complete mitochondrial genomes of two freshwater snails provide new protein-coding gene rearrangement models and phylogenetic implications

**DOI:** 10.1186/s13071-017-2287-1

**Published:** 2017-07-21

**Authors:** Xidong Mu, Yexin Yang, Yi Liu, Du Luo, Meng Xu, Hui Wei, Dangen Gu, Hongmei Song, Yinchang Hu

**Affiliations:** Key Laboratory of Tropical&Subtropical Fishery Resource Application & Cultivation, Ministry of Agriculture, Pearl River Fisheries Research Institute, Chinese Academy of Fishery Sciences, Xingyu Road1, Guangzhou, 510380 China

## Retraction

The authors are retracting this article [1]. A reader recently raised questions related to the identification of one of the snail species whose complete mitochondrial (mt) genomes have been characterised in our article, because the gene order and mt genome sequence of the sample of *Radix swinhoei* (family Lymnaeidae) strongly resemble those of *Physella acuta* (family Physidae) (GenBank JQ390525.1 and JQ390526.1) published by Nolan et al. [2].

The initial morphology-based identification of the snail as “*Radix swinhoei*” was not tested with BLAST searches and as a result we did not realise that we had characterised the mitochondrial genome of a different species. Upon re-examination of our data, we suggest that the sample “*Radix swinhoei*” does in fact represent a species of *Physella* (referred to as *Physella* sp.). Because of this misidentification and the fact that the phylogenetic analysis did not include members of the family Physidae, the conclusions drawn from the “*Radix swinhoei*” sample in our article are incorrect.

All authors agree with this retraction.
